# New York News

**Published:** 1856-09

**Authors:** 


*New York News.*—The place in the Thirteenth Street School, made vacant by the resignation of Prof. Edward H. Parker, is not yet filled.

As we anticipated, Prof. St. John, the able Professor of Chemistry in the Cleveland Medical College, has been appointed to the chair of Chemistry in the College of Physicians and Surgeons. This deprives the Cleveland school of another of its principal props.

The New York Medical Times is dead. The New York Journal of Medicine bought its subscription list. Since that event, Drs. Purple and Smith have disposed of the proprietorship of the Journal to their publishers.

Prof. Chandler R. Gilman is superintending a new edition of Beck's Materia Medica in behalf of the widow of Prof. Beck, as we understand. We trust it may have, what it so well deserves, a large circulation.

